# A Rare Case of Atypical Haemolytic Syndrome Following Right Retrograde Intrarenal Surgery (RIRS)

**DOI:** 10.7759/cureus.86585

**Published:** 2025-06-23

**Authors:** Antonio Cretì, Francesco Pinto, Maria Chiara Sighinolfi, Bernardo Maria Cesare Rocco, Domenico Nigro, Mauro Ragonese

**Affiliations:** 1 Department of Urology, Policlinico Universitario Fondazione Agostino Gemelli, Istituto di Ricovero e Cura a Carattere Scientifico (IRCSS), Rome, ITA

**Keywords:** atypical hemolytic uremic syndrome, eculizumab, retrograde intrarenal surgery, septic shock, surgical complications

## Abstract

Renal failure, non-immune hemolytic anemia, and thrombocytopenia are the hallmarks of the uncommon but potentially fatal complement system-related illness known as atypical hemolytic uraemic syndrome (aHUS). We describe the case of a 46-year-old woman who underwent right retrograde intrarenal surgery (RIRS) for a 1.5 cm urinary stone in the renal pelvis and experienced septic shock on postoperative day 1 caused by aHUS. The patient received continuous venovenous hemodiafiltration (CVVHDF) for six days, eculizumab, a monoclonal antibody that blocks terminal complement activation, and three plasma exchanges with fresh frozen plasma, resulting in a progressive normalization of hemolytic parameters. Two months after the RIRS, the patient underwent percutaneous nephrolithotripsy to remove residual kidney stones. Three days before the surgery, she received a dose of eculizumab. The procedure went smoothly, and there were no postoperative complications. To the best of our knowledge, this is the report that mentions aHUS as a postoperative complication following endourology procedures. This report focuses on multidisciplinary diagnosis, treatment, and follow-up strategies for this unique complication in urology.

## Introduction

Atypical hemolytic uraemic syndrome (aHUS) is a rare and life-threatening disorder characterized by the triad of acute renal failure, non-immune hemolytic anemia, and thrombocytopenia. It is primarily associated with dysregulation of the complement system, often triggered by genetic predispositions or external factors such as infections, medications, or surgery [1,2]. The condition is distinct from other thrombotic microangiopathies due to its underlying complement-mediated pathophysiology and often requires targeted therapeutic interventions, including complement inhibitors like eculizumab [3,4].

Although aHUS is most commonly reported in systemic illnesses or post-transplant settings, its occurrence as a postoperative complication following urological procedures remains exceedingly rare. Here, to the best of our knowledge, we describe the first documented case of aHUS occurring after retrograde intrarenal surgery (RIRS), highlighting the challenges in diagnosis, the multidisciplinary approach to management, and the strategies employed to prevent recurrence during subsequent procedures.

## Case presentation

**Figure 1:**
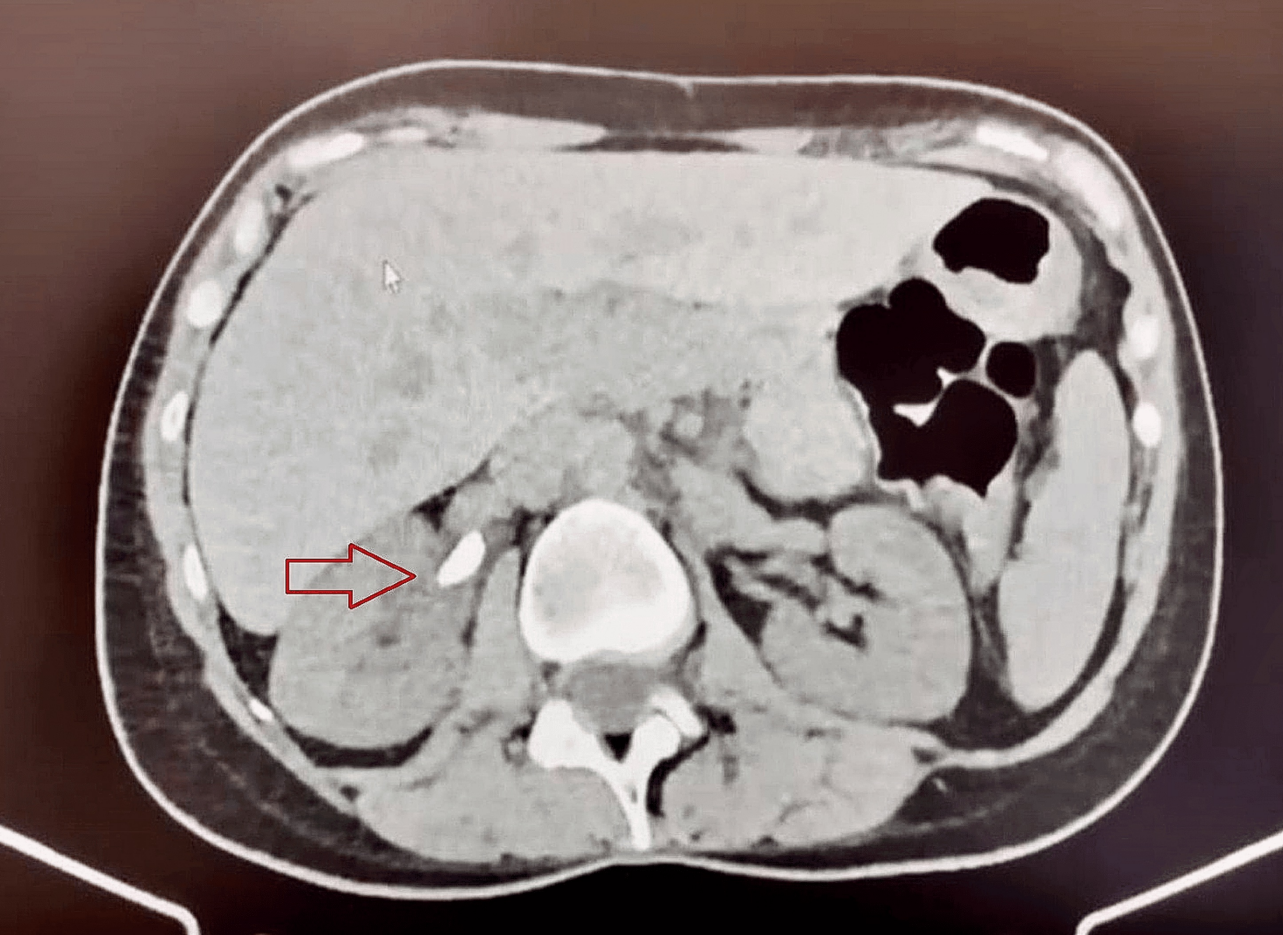
Preoperative computed tomography scan showing a 1.5 cm spindle-shaped urinary stone in renal pelvis

A 46-year-old female patient presented with a history of symptomatic recurrent nephrolithiasis, never treated surgically, starting in 2021. She underwent a follow-up ultrasound in 2022, which showed a 2 cm right renal pelvic stone with mild proximal pyeloureteral ectasia. The patient had recurrent colic pain, but the renal stones were passed spontaneously. She visited our hospital in February 2024 and underwent a CT scan, which revealed a 1.5 cm spindle-shaped calcium oxalate stone in the renal pelvis (Figure 1). She underwent right RIRS three months later on May 24, 2024. Preoperative assessments, including renal function tests and imaging, were unremarkable. The last colic episode occurred approximately eight months before the RIRS procedure.

The patient’s medical history included several relevant factors: her father had hypercholesterolemia, type 2 diabetes mellitus, and an undiagnosed cardiac arrhythmia, while her mother was in good health. Her sister, aged 40, had two spontaneous miscarriages in the past year despite normal genetic testing. Regarding the obstetric anamnesis, the patient experienced two premature deliveries, one at 29+5 weeks and another at 36 weeks, due to uterine vascular insufficiency with fetal growth restriction caused by pre-eclampsia. Nonetheless, her menarche was at age 12 with regular menstruation. Other comorbidities reported are hypothyroidism diagnosed 11 years ago following the first pregnancy, and hypercholesterolemia, untreated. Additionally, the patient underwent appendectomy, two cesarean sections, and cholecystectomy four years prior to the first symptoms of nephrolithiasis.

The RIRS procedure was uneventful. The surgery lasted one and a half hours; 80% of the pelvic renal stone was cleared, and a second endoscopic procedure was scheduled. The immediate postoperative course was also uneventful. On postoperative day (POD) 1, the patient experienced a sudden onset of fever with chills (up to 39.5°C), hypotension, and oliguria (100 cc of urine in 12 hours), progressing to septic shock. Laboratory findings included: acute kidney injury with a creatinine level of 2.6 mg/dL, features of thrombotic microangiopathy, including oliguria, hemoglobin drop (from 14g/dl to 8 g/dl) with consumed haptoglobin <10 mg/dl (reference level 30-200), and thrombocytopenia (platelet nadir at 23,000/µL). Direct Coombs test was negative. Blood and urine cultures were sent, and the first dose of piperacillin-tazobactam 2.25 g was administered. Despite initial stabilization efforts, the patient’s condition deteriorated, necessitating transfer to the intensive care unit (ICU).

ICU management

A diagnosis of aHUS was suspected based on the clinical triad and confirmed through complement assays, which revealed significantly reduced complement factor H activity [2]. The evolution of hemolysis markers during the hospital stay is shown in Table 1.

**Table 1:** Laboratory findings for hemolysis markers and complement levels LDH: lactate dehydrogenase

Timepoint	LDH (U/L)	Haptoglobin (mg/dL)	Reticulocytes (%) of circulating RBCs	Complement Factor H (mg/dL)	C3 (mg/dL)	C4 (mg/dL)
Reference Range	135–225	30–200	0.5–2.5	116–562	90–180	10–40
Pre-operative	180	45	1,2	Normal	Normal	Normal
Postoperative Day 1	620	<10	3,5	Decreased	Low	Normal
ICU Day 1	750	<10	4,2	Significantly reduced	Low	Normal
ICU Day 2	700	<10	4	Reduced	Low	Normal
ICU Day 6	400	15	2,5	Improving	Low	Normal
Post-ICU Day 4	250	35	1,8	Normalizing	Near Normal	Normal

To establish a differential diagnosis, the patient underwent ADAMTS13 activity testing and was evaluated by hematology and nephrology consultants. Initial management included therapeutic plasma exchange and the administration of eculizumab. On May 26, 2024, on POD 2, the second posta plasma exchange session was performed, with 3000 mL of plasma removed and replaced with 15 units of pharmaceutical-grade plasma. Post procedure, the patient received 900 mg of eculizumab in 180 mL of saline over 40 minutes. Concurrently, corticosteroid therapy with methylprednisolone 40 mg twice daily was initiated.

On POD 3, ADAMTS13 activity results indicated 75%, effectively excluding thrombotic thrombocytopenic purpura (TTP). As a result, further plasma exchange was discontinued. During hospitalization, the patient was vaccinated against encapsulated bacteria, including Bexsero (meningococcus B), Nimenrix (tetravalent meningococcus), Prevenar 13 (pneumococcus), and Act-HIB (*Haemophilus influenzae* type B).

In the ICU, the patient received intensive care for septic shock and multiorgan dysfunction. Management included: (i) hemodynamic support: administration of noradrenaline to maintain mean arterial pressure above 65 mmHg, alongside judicious fluid resuscitation to optimize perfusion [3]; (ii) antibiotic therapy: empirical broad-spectrum antibiotics were tailored to cover common uropathogens and adjusted based on clinical response and negative culture results. Infectious disease specialists ruled out bacterial sepsis as a trigger for the patient’s symptoms; (iii) renal support: continuous renal replacement therapy (CRRT) with CVVHDF was initiated to address acute renal failure and maintain electrolyte and acid-base balance [3]. In collaboration with hematology specialists, the team initiated a comprehensive workup for thrombotic microangiopathies, leading to the confirmation of aHUS. Infectious triggers were considered but excluded after extensive evaluation [5].

The therapeutic approach focused on managing the complement-mediated pathology of aHUS and included: (i) complement inhibition: eculizumab was administered promptly after diagnosis. The patient received the initial dose as a loading infusion, followed by maintenance doses according to established protocols. Serial measurements of complement activity guided therapy [4]; (ii) plasma therapy: three sessions of plasma exchange with fresh frozen plasma were performed over a one-week period. This intervention facilitated the removal of circulating complement components and provided functional complement regulators [6]; (iii) renal support continuation: CRRT was maintained for six days until renal function improved, allowing transition to intermittent hemodialysis [3].

**Figure 2:**
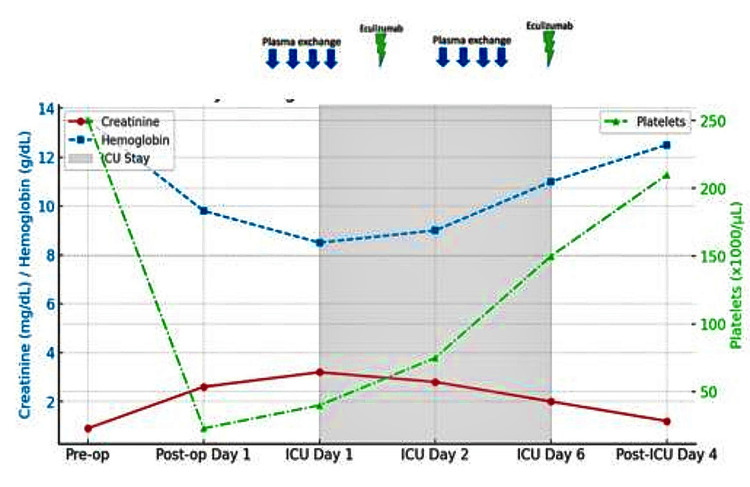
Laboratory trends in creatinine, hemoglobin levels and platelet counts before, during, and after ICU admission

By POD 10, the patient demonstrated significant clinical improvement, with normalization of hemoglobin levels, platelet counts, and creatinine levels, as reported in Figure 2.

To address the remaining renal calculi, the patient underwent stone-free right percutaneous nephrolithotripsy (PCNL) in July 2024, two months after the RIRS. Prophylactic administration of eculizumab three days before the procedure effectively prevented recurrence of aHUS. The PCNL was completed without intraoperative or postoperative complications. The patient remained asymptomatic during follow-up visits, with stable renal function and no signs of recurrent thrombotic microangiopathy [1,4].

## Discussion

This case highlights the critical need for prompt recognition and management of aHUS as a postoperative complication, particularly in the context of endourological procedures. Although there are some reports of postoperative aHUS developed after laparoscopic surgery and myomectomy [6], to our knowledge, this is the first described case of aHUS after endoscopic surgery.

The clinical presentation of renal failure, hemolytic anemia, and thrombocytopenia in the postoperative setting necessitates a high index of suspicion for thrombotic microangiopathies, including aHUS [2,4]. This case underscores the diagnostic and therapeutic challenges associated with postoperative aHUS and the critical role of complement inhibition in improving patient outcomes.

The development of aHUS in the current patient following an otherwise uneventful RIRS suggests that surgical procedures may serve as a trigger for complement system dysregulation in genetically predisposed individuals. Similar to previously reported cases [1], our patient developed acute kidney injury, microangiopathic hemolytic anemia, and thrombocytopenia, necessitating urgent complement inhibition therapy with eculizumab. However, unlike other contexts where aHUS presents in a setting of systemic inflammation or endothelial injury due to infections or autoimmune disease [7], our case uniquely followed a minimally invasive, endoscopic procedure with no intraoperative complications, suggesting that local renal trauma or hemodynamic stress may serve as an underrecognized trigger for complement activation in predisposed individuals. Furthermore, while many reported cases of aHUS in the literature describe a subacute onset and delayed diagnosis, our patient exhibited rapid clinical deterioration within 24-48 hours postoperatively, underscoring the need for high suspicion even in low-risk surgical contexts. 

This case expands the clinical spectrum of aHUS triggers and emphasizes the importance of recognizing thrombotic microangiopathy even after non-transplant genitourinary interventions, especially when hematological abnormalities emerge rapidly postoperatively.

The activation of the alternative complement pathway, often due to genetic mutations or acquired factors, leads to endothelial damage, microvascular thrombosis, and subsequent multiorgan dysfunction [8]. Although infections and ischemic injury are well-established precipitants of aHUS, the potential role of surgical trauma and hemodynamic alterations in precipitating the disease warrants further investigation.

The successful use of eculizumab underscores its pivotal role in treating complement-mediated aHUS and preventing recurrence in high-risk scenarios. This aligns with findings from previous reports of aHUS in surgical and transplant settings, such as a de novo episode following kidney transplantation [9] and postoperative cases triggered by infectious complications [10].

## Conclusions

This report documents a rare case of aHUS as a postoperative complication of retrograde intrarenal surgery, emphasizing the importance of multidisciplinary collaboration in diagnosis, treatment, and follow-up care. The prophylactic use of complement inhibitors represents a promising strategy for preventing recurrence in patients undergoing subsequent urological interventions. Further studies are needed to explore the pathophysiological mechanisms linking surgical triggers to complement activation in aHUS.
